# Quantitative Prediction of Protein Content in Corn Kernel Based on Near-Infrared Spectroscopy

**DOI:** 10.3390/foods13244173

**Published:** 2024-12-23

**Authors:** Chenlong Fan, Ying Liu, Tao Cui, Mengmeng Qiao, Yang Yu, Weijun Xie, Yuping Huang

**Affiliations:** 1 College of Mechanical and Electronic Engineering, Nanjing Forestry University, Nanjing 210037, China; fancl@njfu.edu.cn (C.F.); liuying@njfu.edu.cn (Y.L.); weijun_xie@njfu.edu.cn (W.X.); huangyuping@njfu.edu.cn (Y.H.); 2 College of Engineering, China Agricultural University, Beijing 100083, China; cuitao@cau.edu.cn; 3 Key Laboratory for Theory and Technology of Intelligent Agricultural Machinery and Equipment, Jiangsu University, Zhenjiang 212013, China; yu_yang@ujs.edu.cn

**Keywords:** corn powder, near infrared, spectral imaging, predictive models, machine learning

## Abstract

Rapid and accurate detection of protein content is essential for ensuring the quality of maize. Near-infrared spectroscopy (NIR) technology faces limitations due to surface effects and sample homogeneity issues when measuring the protein content of whole maize grains. Focusing on maize grain powder can significantly improve the quality of data and the accuracy of model predictions. This study aims to explore a rapid detection method for protein content in maize grain powder based on near-infrared spectroscopy. A method for determining protein content in maize grain powder was established using near-infrared (NIR) reflectance spectra in the 940–1660 nm range. Various preprocessing techniques, including Savitzky−Golay (S−G), multiplicative scatter correction (MSC), standard normal variate (SNV), and the first derivative (1D), were employed to preprocess the raw spectral data. Near-infrared spectral data from different varieties of maize grain powder were collected, and quantitative analysis of protein content was conducted using Partial Least Squares Regression (PLSR), Support Vector Machine (SVM), and Extreme Learning Machine (ELM) models. Feature wavelengths were selected to enhance model accuracy further using the Successive Projections Algorithm (SPA) and Uninformative Variable Elimination (UVE). Experimental results indicated that the PLSR model, preprocessed with 1D + MSC, yielded the best performance, achieving a root mean square error of prediction (RMSEP) of 0.3 g/kg, a correlation coefficient (R_p_) of 0.93, and a residual predictive deviation (RPD) of 3. The associated methods and theoretical foundation provide a scientific basis for the quality control and processing of maize.

## 1. Introduction

The global food security crisis is exacerbated by the rise in the world’s population and the escalating demand for key commodities like corn [1]. Corn, one of the world’s important food crops, requires important quality control throughout its production and processing [2,3]. Corn is the largest grain crop in China, and it is also an important component of animal feed and industrial raw materials [4]. The nutritional content of corn, especially its protein content, directly affects its application value in food and feed [5]. Therefore, accurately detecting the protein content of corn grains is crucial for improving the utilization efficiency of corn and ensuring food safety.

Although they have high accuracy, the operation of the traditional methods of protein content determination, such as Kay’s nitrogen determination and the Dumas method [6], is complex, time-consuming and requires the use of a large number of chemical reagents. This limits their application in the production process [7]. In recent years, with the advancement of analytical technologies, near-infrared spectroscopy (NIR) has gradually become an essential means for component analysis in grains and their processed products due to its advantages of speed, non-destructiveness, and environmental friendliness [8].

Near-infrared technology is based on the phenomenon of light absorption caused by molecular vibration and rotation. This technique can obtain spectral information from samples quickly and accurately [9]. By analyzing spectral data, one can infer the chemical composition and content of the samples [10]. Many researchers have recently applied NIR technology to the composition analysis of grains and their processed products, achieving significant results [11]. For instance, Wang et al. conducted online predictions of protein content in corn seeds based on NIR spectroscopy and machine learning, realizing both qualitative and quantitative analysis of corn seeds [12]. Zhang et al. designed a real-time online detection system for protein content in grains during the harvest using a near-infrared spectroscopic combined harvester [13]. Wu et al. employed NIR spectroscopy combined with A-CARS-PLS (Anchor competitive adaptive reweighted sampling) to determine protein content in corn [14]. An optimization strategy for waveband selection of corn protein was also developed based on Fourier-transform near-infrared (FT-NIR) spectrometry [15]. The results indicate that FT-NIR spectrometry combined with waveband optimization technology is promising as an alternative method for detecting the chemical composition of corn.

However, despite previous studies indicating the potential of NIR technology for detecting protein content in corn, challenges remain [16]. NIR technology faces limitations due to surface effects and sample uniformity when measuring the protein content of intact corn grains [17]. This means that the method may be affected by light scattering at the grain surface, leading to inconsistent measurement results. Additionally, corn grain samples’ poor uniformity may influence spectral measurements’ repeatability and accuracy [18]. Using powdered corn grain samples offers better uniformity and can provide consistent spectral signals, thereby enhancing the accuracy and repeatability of detection. Furthermore, powdered samples facilitate sufficient contact with near-infrared light, increasing the spectral response [19]. Thus, further NIR technology exploration and optimization for detecting protein content in corn grain powders hold significant theoretical and practical value.

Therefore, this study aims to develop a rapid and accurate method for quantitative analysis of protein content in powdered corn grains based on near-infrared spectroscopy. The research objectives are as follows: (1) to compare the effects of different preprocessing techniques (multivariate scattering correction, standard normal variate, Savitzky−Golay smoothing, Savitzky−Golay smoothing combined with the first derivative (1D + SG), multivariate scattering correction combined with the first derivative (1D + MSC), standard normal variate combined with the first derivative (1D + SN)) and modelling methods (partial least squares regression, support vector machine, and extreme learning machine regression) on the prediction performance based on the full wavelength; (2) to use UVE and the SPA for feature variable selection; and (3) to optimize the prediction model. The relevant research aims to provide a scientific basis for the quality control of corn.

## 2. Materials and Methods

### 2.1. Preparation of Corn Seed Powder

**Figure 1:**
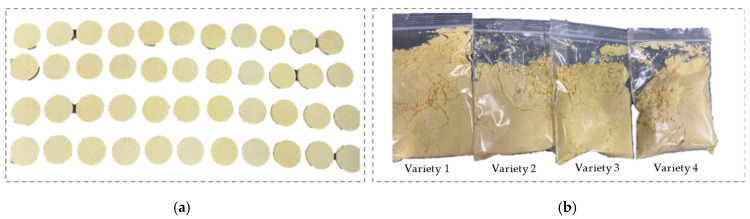
Physical images of powdered maize grain prepared from different varieties: (**a**) the prepared corn grain powder; (**b**) the kernels powder samples of different maize varieties.

To improve the universality of the samples, corn grain powder from different harvesting periods and varieties was collected. The samples originated from five major production areas: Northeast, North China, Huang-Huai-Hai, Southwest, and Northwest. To reduce the influence of unrelated external factors in the experiment, this study involved washing and drying the corn kernels before grinding. Fresh, unspoiled corn grains were selected and cleaned to remove impurities. After washing, the corn kernels were dried in a drying oven (GZX-9140MBE, Shanghai, China) at 50–60 °C. Then, the moisture content of corn grain powder was determined by water detector (XIUILAB MB27, Shanghai, China) to be 13.05%. This already met the requirements of Chinese national standards, and the moisture content of dried corn grain should be maintained between 13% and 14% to ensure the safe storage and quality of the corn. The step helped eliminate the impact of the spectral characteristics of moisture absorption on the near-infrared spectroscopy detection of protein. Then, a high-speed grinder (AZL 4500A, Jinhua, China) was used to grind the dried corn kernels to obtain the fine powder. The ground corn powder was then filtered through an 80-mesh sieve for subsequent physicochemical measurements. The resulting corn grain powder sample is shown in Figure 1. Ninety-two sample groups were prepared, with each group randomly selecting three collection points for near-infrared spectroscopy information collection.

### 2.2. Near-Infrared Detection System for Protein Content

**Figure 2:**
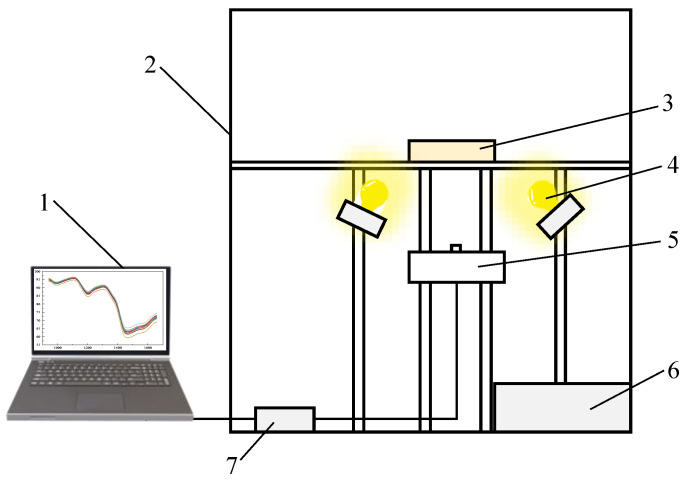
Protein content detection platform based on near-infrared spectroscopy. 1. Computer; 2. camera obscura; 3. corn kernel powder sample; 4. illuminant; 5. optical fiber; 6. power source; 7. spectrograph.

A near-infrared spectroscopy detection system was constructed to facilitate the collection of spectral reflectance information from corn grain powder (Figure 2). The detection system mainly consists of a light source, optical fibers, a sample stage, and a spectrometer. The FLAME-NIR-INTSMA25 spectrometer (Ocean Optics, Dunedin, FL, USA) was selected, which has a detection wavelength range of 940 to 1660 nm. By avoiding the visible light wavelength range, the system effectively minimizes the influence of the corn powder’s color on the overall protein content prediction.

### 2.3. Spectral Information-Preprocessing Method

Since the detection platform is a relatively open spectroscopic detection system, during the collection of sample spectral information, the corn powder’s spectral information is captured, along with some random noise, stray light, electrical noise, and other unrelated external information [20]. Therefore, it is essential to apply appropriate spectral preprocessing algorithms to process the raw spectra before conducting spectral data analysis.

Common spectral preprocessing algorithms include standard normal variate transformation (SNV), derivatives, smoothing, multiplicative scatter correction (MSC), and baseline correction [21,22]. In this study, based on the powdered nature of corn powder and the volume of spectral data, six different algorithms were selected as preprocessing methods: Savitzky-Golay (S-G) convolution smoothing, multiplicative scatter correction (MSC), standard normal variate transformation (SNV), the first derivative (1D), the first derivative combined with S-G smoothing (1D + SG), the first derivative combined with multiplicative scatter correction (1D + MSC), and the first derivative combined with standard normal variate transformation (1D + SNV).

(1) Multiplicative scatter correction (MSC)

Multiplicative scatter correction (MSC) can eliminate the light scattering effects caused by uneven particle size distribution in samples, thereby reducing the impact of the inherent non-uniformity of corn powder samples on the spectral information [23]. The principle involves establishing a linear equation between the spectral reflectance at each wavelength for each sample and the average spectral reflectance at that wavelength. The intercept reflects the sample-specific reflection information, while the slope indicates the uniformity of the sample. The final multiplicative scatter corrected spectrum is calculated using the following formula, aiming to minimize random variations to the greatest extent possible:

$$x_{MSC}=\frac{x_{i}-b_{o}}{b}$$

where *x_i_* is the spectral curve of the *i*th sample, *b_o_* is the intercept between the *i*th sample spectrum and the average spectrum, and *b*th is the slope between the *i* sample spectrum and the average spectrum.

(2) Standard normal variate transformation (SNV)

Standard normal variate transformation (SNV) functions are similar to multiplicative scatter correction (MSC) and is primarily used in spectral detection to eliminate the effects of solid particle size, surface scattering, and variations in optical path length on reflectance spectra [24]. Therefore, it is expected to improve the spectral data of corn powder effectively. During the correction process, each spectrum is handled individually, assuming that the absorbances at various wavelengths within the same spectrum follow a certain distribution. Standard spectra are not required; instead, the original spectrum is adjusted by subtracting its overall mean and dividing by the spectral data’s standard deviation (*σ*). This process results in a processed spectral curve as follows:

$$x_{SNV}=\frac{x_{i}-\bar{x}}{\sigma }$$

where *x_i_* is the spectral curve of the *i*th sample, *σ* is the standard deviation, and $\bar{x}$ is the overall spectral average

(3) Savitzky−Golay convolution smoothing (S−G)

Savitky−Golay smoothing was proposed by Savitky and Golay in 1964. The least-square fitting coefficient is introduced to smooth the original spectrum [25]. It is mainly used for low-pass filtering, eliminating high-frequency components, and achieving the purpose of retaining effective low-frequency information and improving the signal-to-noise ratio. In this study, according to the pixel count of the spectrometer and relevant experience, the S−G smoothing number was selected as 5, and the original spectrum was smoothed at 5 points. The following formula can calculate the smoothed average value at wavelength *λ*:

$$x_{\lambda ,smooth}=\frac{1}{H}\sum_{i=-w}^{+w}x_{\lambda_{i+1}}h_{i}$$

$$H=\sum_{i=-w}^{+w}h_{i}$$

where *h_i_* is the smoothing coefficient and *H* is the normalization factor.

(4) The first derivative (1D)

Derivative preprocessing in near-infrared spectral detection can help eliminate baseline drift and reduce background interference, resulting in clearer spectral curves [26]. Precisely, first derivative preprocessing can remove baseline variations that are independent of wavelength. The spectra processed using this method better reflect the changing trends of the original spectra and can also provide a certain level of separation for characteristic peaks affected by interference effects. However, the differentiation process can also introduce noise, which may affect the analysis of the intrinsic characteristic information of the samples. Therefore, in addition to the first derivative processing, this study employs MSC, SNV, and S−G methods for preprocessing to provide corresponding compensation based on the first derivative.

### 2.4. Feature Screening Method

Near-infrared spectroscopy is a technique that predicts the content and composition of substances based on the structure and content information of X-H groups. However, the spectrum contains not only the quality information of the object to be measured but also multicollinearity and redundant information between continuous wavelengths, leading to high computational demands and slow processing speeds [27]. Therefore, this study employs the Successive Projections Algorithm (SPA) and Elimination of Uninformative Variables (UVE) to extract characteristic wavelengths related to the fundamental properties of corn protein from the entire spectral range. This approach aims to reduce interference from irrelevant noise or variables, enhance the model’s predictive capability and improve its interpretability.

### 2.5. Modeling Method of the Prediction Model

In near-infrared spectral analysis, associating sample spectral information with its related detection parameters and establishing the corresponding calibration models is an essential step in predicting the intrinsic properties and composition of the samples to be tested. These analyses can be broadly categorized into qualitative and quantitative analyses based on the identification patterns. Quantitative analysis focuses more on studying the relationship between the material information collected by the spectral system and the concentration of the material components in the samples. Standard regression algorithms include Partial Least Squares Regression (PLSR) [28] and Support Vector Machine Regression (SVM) [29,30,31]. In this study, based on the characteristics of the information collected by the spectrometer and the inherent properties of the corn powder samples, both linear and nonlinear algorithms were selected for model comparison. Specifically, Partial Least Squares Regression (PLSR), Support Vector Machine (SVM), and Extreme Learning Machine Regression (ELM) were chosen as modeling methods to compare their predictive effects.

(1) Partial Least Squares Regression (PLSR)

Partial Least Squares Regression (PLSR) is a commonly used multivariate analysis method in quantitative analysis that decomposes and calculates two data matrices, X and Y, to obtain their score matrices and loading matrices, followed by linear regression. It can analyze the relationships between data matrix X, which exhibits strong multicollinearity, and multiple variable matrix Y [32]. However, in practical detection, factors such as internal light scattering between corn grain powder particles, overlapping characteristic peaks, and external noise interference may lead to a certain level of nonlinearity between spectral variables and concentrations. Therefore, this study also includes nonlinear algorithms for model comparison. The corn powder spectral matrix X and the protein content matrix Y are decomposed.

(2) Support Vector Machine (SVM)

Support Vector Machine (SVM) is a commonly used supervised learning algorithm, mainly used for classification and regression analysis. SVR is a regression analysis method based on Support Vector Machine (SVM), which can map low-dimensional nonlinear input variables to high-dimensional linear spaces. Then, linear regression is formed by finding the optimal interface. The error of all training samples obtained from the optimal interface is minimal [33]. The choice of kernel function and corresponding parameters (penalty coefficient C and gamma) directly affects SVR’s learning and generalization ability. Therefore, this study set the kernel functions as radial basis function (RBF) and linear kernel function. The above two kernel functions are calculated as follows.

$$K(x_{i},x_{j})=\exp(-\frac{{\left\|x_{i}-x_{j}\right\|}^{2}}{2\sigma^{2}})$$

$$K(x_{i},x_{j})=x_{i}\cdot x_{j}$$

where $\left\|x_{i}-x_{j}\right\|$ is the Euclidean distance between the two input vectors, and $\sigma^{2}$ is a parameter that controls the spread of the kernel.

(3) Extreme Learning Machine (ELM)

Extreme Learning Machine (ELM) is a novel learning algorithm for single-hidden-layer feedforward neural networks proposed by Huang [34]. It randomly generates connection weights between the input layer and the hidden layer, as well as the thresholds for the hidden layer neurons, enabling analytical measurement of the weights in the single-hidden-layer feedforward network. Compared to gradient-based learning algorithms, ELM exhibits superior generalization capability and faster execution speed, as well as avoiding the complications associated with parameter selection and local minima.

### 2.6. Evaluation Index

The correlation coefficient (R), root mean square error (RMSE), and relative analysis error (RPD) were used to evaluate the prediction effect of protein content in corn meal. An RPD between 1.5 and 2 means that the model can discriminate low from high values of the response variable; a value between 2 and 2.5 indicates that coarse quantitative predictions are possible, and a value between 2.5 and 3 or above corresponds to good and excellent prediction accuracy, respectively [35]. SD is the standard deviation of the reference values (observed data). The calculations of evaluation indicators are as follows [17]:

$$R=\sqrt{1-\frac{\sum_{i=1}^{n}{\left(y_{i,actual}-y_{i,predicted}\right)}^{2}}{\sum_{i=1}^{n}{\left(y_{i,actual}-y_{average}\right)}^{2}}}$$

$$RMSE=\sqrt{\frac{\sum_{i=1}^{n}{\left(y_{i,actual}-y_{i,predicted}\right)}^{2}}{N-1}}$$

$$RPD=\frac{SD}{RMSE_{P}}$$

## 3. Results

### 3.1. Sample Set Partitioning of Protein Content Prediction Models

In the collected 92 samples, the average protein content was 9.79%, consistent with the typical values for standard corn based on previous research statistical data [36,37]. The protein content ranged from 8.43% to 11.25%, covering most of the composition range of corn kernels, indicating that the selected samples were somewhat representative.

Furthermore, the 92 samples were divided into a calibration set (*n* = 69) and a prediction set (*n* = 23) using a concentration gradient method with a ratio of 3:1 [38]. The reason for splitting the data set into two subsets is that in small data sets, the additional split might lead to a smaller training set which may be exposed to overfitting. To provide enough data for training, the validation set was used to assess the performance of the models [39,40]. The results of the sample division are shown in Table 1. The protein content of the prediction set was included within the calibration set. The ranges and standard deviations of the content were relatively similar, suggesting that the content distributions of the two groups were fairly uniform, thereby avoiding bias in their distributions. Overall, these samples met the basic requirements for modeling.

### 3.2. Near-Infrared Spectral Curve Analysis

**Figure 3:**
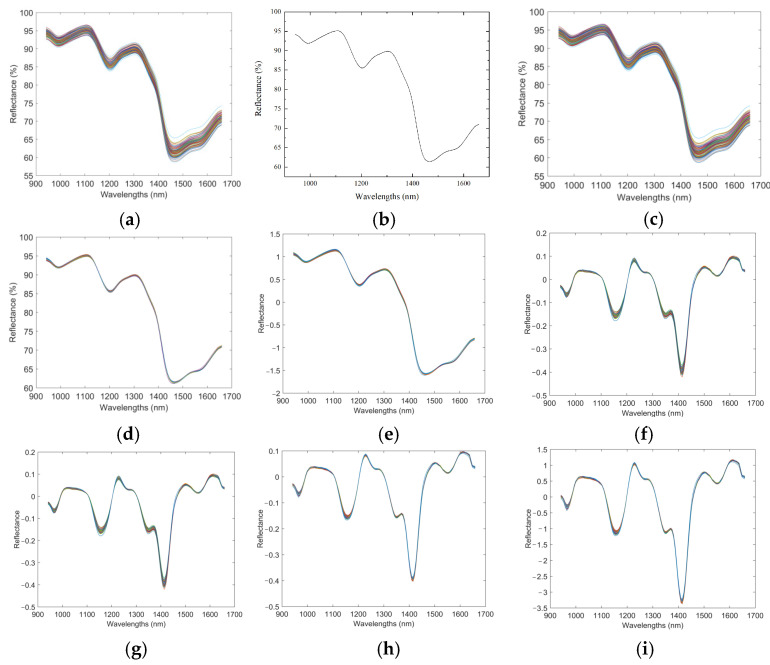
Raw spectral data and preprocessing techniques. (**a**) Spectral curves for all samples; (**b**) average spectral curve across all samples; (**c**) preprocessing with the Savitzky−Golay (SG) method; (**d**) preprocessing with multiplicative scatter correction (MSC); (**e**) preprocessing with standard normal variate (SNV); (**f**) preprocessing using the first derivative (1D); (**g**) combined preprocessing with the first derivative and Savitzky−Golay smoothing (1D + SG); (**h**) combined preprocessing with the first derivative and multiplicative scatter correction (1D + MSC); (**i**) combined preprocessing with the first derivative and standard normal variate (1D + SNV).

Figure 3 shows the spectral curves of all 92 samples. The average spectrum of three points for each sample was taken as the original spectral curve of that sample. The raw spectral curves of the collected corn flour samples are presented in Figure 3a. It was evident that the trends across all samples were consistent, and there were no outlier samples. The spectral reflectance values between 940 nm and 1600 nm exhibited significant variations, particularly between 940 nm and 1500 nm.

The sample spectra were averaged for easier comparison to obtain a representative spectral curve, as shown in Figure 3b. As molecular vibration spectra, the NIR spectra arise from the absorption of X-H bonds, with variations in the absorption peak intensity and position due to different hydrogen-containing groups. This study observed significant absorption peaks for the corn flour spectral curves at 994 nm, 1212 nm, and 1466 nm. The absorption peak around 1000 nm was related to the second overtone of N-H bond stretching vibrations; the absorption peak around 1200 nm corresponded to the second overtone of C-H bond stretching vibrations, while the absorption peak around 1480 nm was associated with the fundamental frequency of O-H bond stretching vibrations. The characteristic bands of N-H bond absorption ranged from 1000 to 1100 nm and from 1420 to 1520 nm [41,42]. The structural characteristics of proteins primarily consisted of the elements C, H, O, and N, with groups like -NH, -NH2, and -COOH present in their long-chain structures. The chemical composition indicated by the absorption above peaks aligned with the chemical components of -NH, -NH2, and -COOH groups found in proteins.

This study conducted various preprocessing methods on the raw data to enhance model accuracy, including Savitzky−Golay (S−G) smoothing, multiplicative scatter correction (MSC), standard normal variate (SNV), and the first-derivative (1D) preprocessing, with the results shown in Figure 3. The figure shows that the curves after 5-point S−G smoothing visually exhibit no significant changes compared to the original images. Since MSC and SNV serve similar purposes in eliminating the effects of factors such as grain particle size, surface scattering, and background noise, the trends of the spectral curves after MSC and SNV preprocessing remain consistent. After applying the first-derivative (1D) preprocessing, the less prominent peak and trough information was amplified; however, this may have also increased the resolution of noise signals. Therefore, smoothing processes based on the first derivative (1D + SG, 1D + MSC, and 1D + SNV) were applied.

However, because the spectral information is composed of the overtone and combination frequencies of numerous vibrational molecular groups, it is impossible to accurately predict the protein content in corn flour based solely on minor spectral curve changes visible to the naked eye. Therefore, chemometric algorithms should be employed to establish relevant prediction models for forecasting protein content.

### 3.3. Analysis of Protein Content Prediction Model Based on Full Band

#### 3.3.1. PLSR Algorithm Model (Linear Algorithm Modeling Method)

The protein content prediction model was constructed according to the correction set and the prediction set divided in Table 1. The correction set and the prediction set were divided according to Table 2. In Matlab (R2020b), a full-wavelength spectral prediction model based on the PLSR algorithm was established for the original spectra and seven kinds of pre-processed spectra.

Table 2 shows that, under the PLSR method, the original spectrum can provide coarse quantitative predictions even without using a pretreatment method. Its R_c_ was 0.90, RMSE_C_ was 0.3 g/kg, R_p_ was 0.90, RMSE_P_ was 0.3 g/kg, and RPD was 2.3. The results showed that the model could be used for quantitative analysis of protein content in corn kernels at an acceptable level. The RPD value was 2.3, which reflected an acceptable prediction quality but fell short of being excellent (coarse quantitative predictions). In addition, the prediction of the pre-treated spectral models also varied to different degrees, and the RPD of all models was greater than 2.0, indicating an acceptable level of prediction. It can be further used to determine protein content in corn grain meal quantitatively. The best prediction model was the PLSR model with 1D + MSC (R_p_: 0.92, RMSE_P_: 0.3 g/kg) and 1D + SNV (R_p_: 0.92; RMSE_P_: 0.3 g/kg) pretreatment. At this point, the RPD of both models was 3. The results show that the model has good prediction accuracy and can be used for subsequent prediction.

#### 3.3.2. SVM Algorithm Model (Linear and Nonlinear Algorithm Modeling)

In this study, two kernel functions, linear (line-SVM) and radial basis (rbf-SVM), were selected to construct the detection model of protein content. Using the grid optimization method, the parameters with the minimum error of the model were selected as the best modeling parameters. Table 3 shows the mesh optimization process of SVM based on two kernel functions. As can be seen from the figure, for line-SVM, the optimal model was determined only by the penalty factor *c*, while the kernel function parameter *g* did not influence the model. For rbf-SVM, the optimal model was determined by the two parameters c and g.

Table 3 showed that the SVM model with a linear kernel function generally outperformed the model with a rbf kernel under different preprocessing methods. For the linear kernel, the optimal preprocessing methods were 1D + MSC and 1D + SNV, with R_c_ and R_p_ both reaching 0.93 and 0.91, RMSE_C_ and RMSE_P_ both at 0.3, and RPD achieving 2.4, indicating coarse quantitative predictions. In contrast, single preprocessing methods (e.g., SG, MSC, and SNV) showed similar model performances (RPD around 2.2), suggesting that these simpler preprocessing approaches provided limited improvement.

By comparison, the rbf kernel models generally had lower RPD values (<2.0), indicating weaker predictive capabilities. Although some models achieved high fitting accuracy (R_c_) (e.g., 1D + MSC with R_c_ = 0.98), their prediction accuracy (R_p_) and RPD were poor (0.84 and 1.9, respectively), showing that the rbf kernel failed to effectively capture the relationship between spectral information and protein content. Therefore, the linear kernel was more suitable for building protein content prediction models, and combining it with 1D + MSC or 1D + SNV preprocessing significantly enhanced model performance.

#### 3.3.3. ELM Algorithm Model (Nonlinear Algorithm Modelling Method)

Table 4 shows the results of the optimal protein content model established based on ELM. The corresponding optimum number of neurons is also shown in Table 4. The results showed that the accuracy of the protein content model constructed after MSC and SNV pretreatment was better than that of the original data, consistent with the results of PLSR and SVM. It was again verified that MSC and SNV were effective pretreatment methods for establishing a protein content model. The accuracy of the protein content model was further improved after the first-order guide and its combination and treatment. The best detection models for protein content were 1D + SNV, R_p_, RMSE_p_, and RPD, which were 0.91, 0.3, and 2.5, respectively. For the protein content detection model processed with 1D + SNV, an RPD value between 2 and 2.5 indicates that the model can provide a coarse quantitative prediction of protein content in the kernels.

In summary, the results of the optimal detection models for protein content based on PLSR, SVM, and ELM indicated that all three modeling methods achieved predictions for protein content in corn flour. Comparatively, the overall predictive performances of ELM and SVM models were weaker than that of the PLSR model. This may be attributed to the inherent characteristics of the ELM algorithms. Because random mapping is performed on the data during execution, the models obtained from multiple iterations are random, resulting in potentially optimal models under given conditions [43]. In the PLSR algorithm, the best preprocessing methods were 1D + MSC and 1D + SNV. The accuracy of the linear SVM algorithm model was generally superior to that of the nonlinear models. Furthermore, the three preprocessing methods—1D + SG, 1D + MSC, and 1D + SNV—all performed well among the linear algorithms. The results suggest that nonlinear methods are more suitable for establishing protein detection models.

Additionally, by comparing the evaluation metrics further, the optimal modeling algorithm was determined by comparing the evaluation indexes (R, RMSE and RPD) values of the rbf-SVM and ELM models. The results indicated that the ELM model developed a better performance than the rbf-SVR model. Therefore, compared to the other algorithms, the PLSR model produced satisfactory results in predicting protein content, especially after first-derivative processing, which yielded the optimal predictive model (good prediction accuracy). Specifically, the preprocessing methods—1D + MSC and 1D + SNV—achieved commendable results. Under the preprocessing conditions of 1D + MSC and 1D + SNV, the evaluation metrics for the PLSR prediction model were as follows: R_p_: 0.92, RMSE_p_: 0.3, RPD: 3 (1D + MSC); and R_p_: 0.92, RMSE_p_: 0.3, RPD: 3 (1D + SNV).

For the protein content detection model, PLSR was the best modeling method. Both 1D + MSC and 1D + SNV were effective preprocessing techniques that can be used for further feature band selection processes. In subsequent research, PLSR will be used as the preferred modeling algorithm for further experimental exploration. This is similar to the results of Zheng et al. and Ye et al. [44,45]. They also demonstrated the effectiveness of PLSR in constructing grain protein prediction models. Compared with other algorithms, the PLSR algorithm can deal with multicollinearity between spectral information better, because it builds the model by extracting the principal component and optimizing the covariance between the independent variable and the dependent variable [44].

### 3.4. Analysis of the Protein Content Prediction Model Based on Characteristic Spectra

The multicollinearity of near-infrared spectral data can decrease model accuracy, making the selection of corresponding spectral features significant for improving model precision. Therefore, reducing the number of variables involved in modeling is crucial for enhancing the software’s operating speed. The author employed various feature selection methods to identify the spectral bands most closely related to protein content, thereby minimizing the number of variables while ensuring model accuracy. Based on the above analysis, the following feature selections were all performed using effective preprocessing methods combined with the best modeling method, PLSR. The results of these processing methods were then compared with the original data to evaluate the effectiveness of the selected features.

#### 3.4.1. Analysis of Feature Filtering Results Based on the Continuous Projection Algorithm (SPA)

The PLSR models established using feature bands selected by the SPA under different spectral preprocessing conditions demonstrated good predictive performance (Table 5). The number of feature bands was reduced from 128 to between 7 and 20. Compared to the original full-band spectral information, the SPA effectively selected features from the original bands, decreasing the feature count to 20 while improving model accuracy to a certain extent. Under the 1D + SNV preprocessing condition, the PLSR model established using feature bands selected by SPA exhibited a slight decrease in performance. This may be due to removing certain feature bands related to protein content, which impacted the model’s predictive capabilities. Overall, the results from the SPA indicated that it reduced the number of feature bands and maintained a certain level of model accuracy. Therefore, for the protein content detection model, the best-performing model was established using feature bands selected by the SPA after the 1D + MSC preprocessing, with R_p_, RMSE_p_, and RPD values of 0.91, 0.3, and 2.4, respectively (*R_c_* = 0.92). This indicates that the model has the ability to provide a coarse quantitative prediction of corn kernel protein content. The optimal number of feature bands selected by SPA was 16.

#### 3.4.2. Analysis of Feature Filtering Results Based on the Uninformative Variable Elimination (UVE)

**Figure 4:**
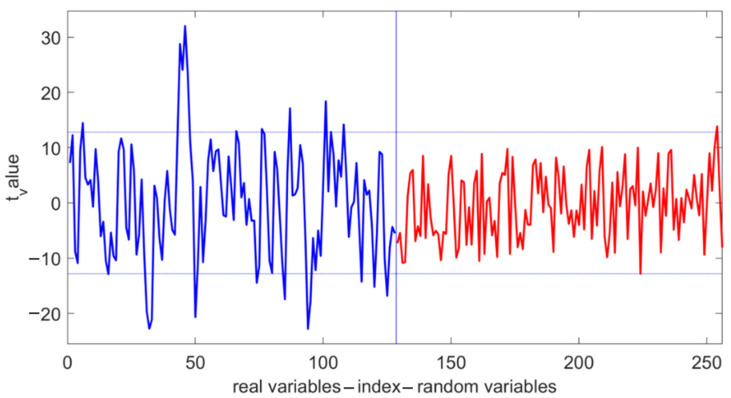
The screening results of the UVE under the optimal model.

The number of feature bands selected by the UVE was generally greater than the corresponding number after feature selection using the SPA (Table 6). Many researchers have also reported similar results [26,38]. This outcome is attributed to the differing principles of the two algorithms. The UVE is primarily used to eliminate irrelevant information, while the SPA is focused on selecting relevant information. That is, the UVE removes irrelevant information, allowing the focus to shift to more significant variables, while the SPA directly selects bands relevant to prediction, keeping the model simpler. In this study, the model accuracy established after feature selection using the UVE showed improved R_p_, RMSE_p_, and RPD values compared to the accuracy established after feature selection using the SPA for this specific instance. Notably, the SPA model also showed an acceptable performance under 1D + MSC pretreatment. R_p_, RMSE_p_, and RPD values were 0.91, 0.3, and 2.4, respectively. Under the 1D + MSC preprocessing condition, the PLSR model established after feature selection by UVE demonstrated significant performance improvement (R_p_ = 0.93; an RPD value of 3 indicated that the model had good prediction accuracy regarding corn kernel protein content). Compared to the SPA algorithm, the UVE retained more characteristic bands that appear to be linked to protein content, which may explain the model’s improved predictive performance. The characteristic wavelength distribution obtained by screening under this model is shown in Figure 4.

**Figure 5:**
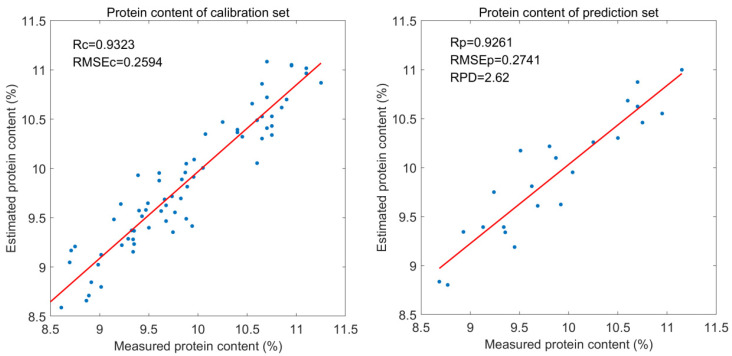
Scatter plots of protein content correction set and prediction set results based on the UVE optimal treatment.

The horizontal axis in the figure represents the wavelength index, with vertical lines serving as variable separation markers. The first 128 indices correspond to the original spectral wavelengths, while the subsequent 128 indices represent randomly generated noise wavelengths. The vertical axis indicates wavelength stability. Two horizontal lines represent the threshold values of ±h_max used as the decision criteria. If the stability of the spectral variables to the left of the vertical line exceeds the positive threshold or falls below the negative threshold, those wavelengths are selected as modeling wavelengths; conversely, they are considered useless and removed. Based on this criterion, the feature wavelengths for each preprocessing method were obtained. As shown in Figure 4, under the 1D + MSC preprocessing, the number of variables participating in modeling decreased from 128 to 23 after UVE selection, achieving the goal of simplifying the complex model. The correction set and the prediction set of the optimal model corresponded to the predicted results of capsaicin content, as shown in Figure 5.

As shown in Figure 5, there was an obvious linear relationship between the true value and the predicted value of grain powder protein content in both the correction and prediction sets, indicating that the model had a high predictive ability. The PLSR model established after feature band selection by the UVE had the best performance. Therefore, this study developed a predictive model using near-infrared technology and machine learning algorithms to achieve accurate detection of protein content in corn kernels. The accuracy of the predictive model is comparable to that of the model constructed based on hyperspectral technology [17]. In terms of economy and convenience, the near-infrared detection technology for corn protein proposed in this paper is superior to the hyperspectral technology.

### 3.5. Comparative Analysis of Feature Screening Results

**Figure 6:**
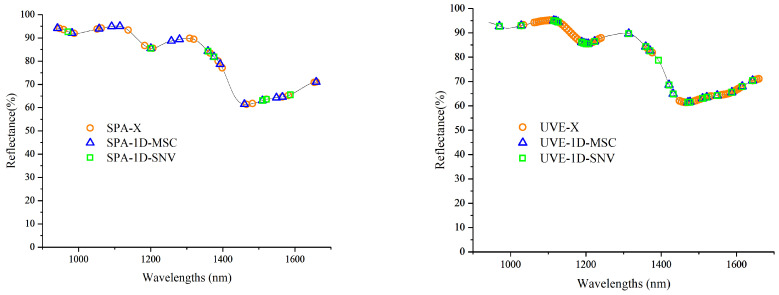
Distributions of characteristic wavelengths screened by the SPA and UVE.

The best characteristic wavelengths related to protein content selected based on the SPA and the UVE (Figure 6). From Figure 6, it can be observed that the characteristic wavelengths selected by the SPA and the UVE were generally consistent. In the near-infrared region, UVE selected the wavelengths of 1194.78 nm, 1206.18 nm, 1471.17 nm, and 1476.75 nm, while the SPA algorithm did not select these four wavelengths. The wavelengths of 1194.78 nm and 1206.18 nm are related to the second harmonic vibrations of the C-H bonds in proteins, and the wavelengths of 1471.17 nm and 1476.75 nm are associated with the first harmonic vibrations of the O-H bonds in proteins [36]. This explains why the protein content model established using the characteristic wavelengths selected by UVE is superior to the model built using the wavelengths selected by the SPA. Furthermore, under the two preprocessing conditions of 1D-MSC and 1D-SNV, the characteristic wavelengths selected by UVE were very similar. However, there were significant differences in the characteristic wavelengths selected by the SPA under the two preprocessing conditions. This further indicated that the UVE feature selection performance was relatively stable. In summary, the characteristic wavelengths selected by the UVE method were reliable and stable. However, without preprocessing, the SPA provides a slightly better performance than UVE. Although the SPA selected fewer bands, it still produced acceptable predictive models, especially with 1D + MSC preprocessing (RPD = 2.4). Moreover, the computational efficiency of SPA was higher because it selected fewer bands, thus reducing the complexity of the model.

## 4. Conclusions

This study utilized near-infrared spectroscopy (NIRS) technology to accurately and effectively determine protein content in corn grain powder. The preprocessing methods of 1D + MSC (multiplicative scatter correction) and 1D + SNV (standard normal variate) were effective for corn powder’s near-infrared spectra. Prediction models based on PLSR (Partial Least Squares Regression), SVM (Support Vector Machine), and ELM (Extreme Learning Machine) were developed using full-band spectral data. The PLSR model demonstrated the best predictive performance by comparing evaluation metrics between the models. Additionally, the SPA (Sequential Feature Selection) and UVE (Univariate Feature Selection) methods were used to select spectral bands associated with protein content in corn grains, effectively reducing the number of modeling variables while maintaining satisfactory predictive performance. The results indicated that the optimal prediction model for protein content was established using characteristic variables selected by UVE following 1D + MSC preprocessing (1D + MSC − UVE − PLSR − Protein model). This model achieved a good prediction accuracy with a correlation coefficient (R_p_) of 0.93, a root mean square error of prediction (RMSE_P_) of 0.3 g/kg, and a ratio of performance to deviation (RPD) of 3. This research provides valuable data and theoretical insights for the industrial application of NIRS in precisely determining corn protein content.

This study focuses on corn kernel powder as the research subject, utilizing near-infrared spectroscopy technology combined with machine learning algorithms to achieve accurate prediction of corn kernel protein content. This method effectively solves the issues arising from the limitations of the surface effects of whole corn kernels and sample uniformity, which in turn affect the accuracy of quantitative detection of corn kernel protein using near-infrared spectroscopy. In practical applications, corn is widely cultivated across various regions with numerous varieties. Future research will include more corn samples, taking into account different cultivation regions and diverse corn varieties. With the addition of large datasets, convolutional neural networks and other deep learning algorithms can be explored to establish a protein content prediction model for corn kernels with broader adaptability and higher precision.

## Figures and Tables

**Table 1:** Reference values of protein content in corn grain powder samples.

Modeling Set	Sample Number	Minimum Value (%)	Maximum Value (%)	Mean Value (%)	Standard Deviation (%)
Training set	69	8.43	11.25	9.86	0.7
Test set	23	8.69	11.15	9.87	0.7
All	92	8.43	11.25	9.79	0.7

**Table 2:** Results of the PLSR model based on different preprocessing methods.

Preprocessing Methods	The Number of Optimal Principal Components	R_c_	RMSE_c_ (g/kg)	Rp	RMSE_p_ (g/kg)	RPD
X	11	0.90	0.3	0.90	0.3	2.3
SG	11	0.90	0.3	0.90	0.3	2.3
MSC	10	0.91	0.3	0.91	0.3	2.3
SNV	10	0.91	0.3	0.91	0.3	2.3
1D	8	0.92	0.3	0.90	0.3	2.3
1D + SG	8	0.90	0.3	0.90	0.3	2.3
1D + MSC	8	0.94	0.24	0.92	0.3	3
1D + SNV	8	0.94	0.24	0.92	0.3	3

Note: Savitzky−Golay (S−G) convolution smoothing, multiplicative scatter correction (MSC), standard normal variate transformation (SNV), the first derivative(1D), the first derivative combined with S-G smoothing (1D + SG), the first derivative combined with multiplicative scatter correction (1D + MSC), and the first derivative combined with standard normal variate transformation (1D + SNV).

**Table 3:** SVM modeling results based on different preprocesses.

Preprocessing Methods	Kernel Function	Best *c*	Best *g*	*R_c_*	RMSE_c_	*R_p_*	RMSE_p_	RPD
X	line	64.0000	\	0.91	0.3	0.90	0.3	2.4
SG		64.0000		0.91	0.3	0.90	0.3	2.4
MSC		2.8284		0.92	0.3	0.90	0.3	2.2
SNV		2.8284		0.92	0.3	0.89	0.3	2.2
1D		0.0442		0.90	0.3	0.91	0.3	2.2
1D + SG		0.0625		0.90	0.3	0.91	0.3	2.4
1D + MSC		0.0625		0.93	0.3	0.90	0.3	2.4
1D + SNV		0.0625		0.93	0.3	0.91	0.3	2.4
X	rbf	724.0773	0.0110	0.98	0.16	0.56	0.7	1.0
SG		724.0773	0.0110	0.97	0.16	0.56	0.7	1.0
MSC		90.5097	0.0010	0.86	0.4	0.82	0.4	1.8
SNV		512.0000	0.0010	0.93	0.3	0.84	0.4	1.8
1D		11.3137	0.0028	0.93	0.3	0.91	0.3	2.4
1D + SG		22.6274	0.0020	0.93	0.3	0.91	0.3	2.5
1D + MSC		2.8284	0.0156	0.98	0.14	0.84	0.4	1.9
1D + SNV		2.8284	0.0156	0.98	0.14	0.84	0.4	1.9

**Table 4:** ELM modeling results based on different preprocesses.

Preprocessing Methods	The Optimal Number of Neurons	*R_c_*	RMSE_c_	*R_p_*	RMSE_p_	RPD
X	34	0.87	0.4	0.87	0.4	1.9
SG	38	0.90	0.3	0.87	0.5	1.5
MSC	48	0.94	0.25	0.87	0.4	2.0
SNV	36	0.94	0.3	0.89	0.4	2.0
1D	38	0.92	0.3	0.89	0.3	2.2
1D + SG	37	0.89	0.3	0.89	0.3	2.2
1D + MSC	34	0.91	0.3	0.89	0.3	2.2
1D + SNV	37	0.94	0.24	0.91	0.3	2.5

**Table 5:** PLSR modeling results based on the SPA feature screening.

Preprocessing Methods	Number of Characteristic Bands	Optimal Number of Principal Components	*R_c_*	RMSE_c_	*R_p_*	RMSE_p_	RPD
X	20	12	0.90	0.3	0.91	0.3	2.4
1D + MSC	16	10	0.92	0.3	0.91	0.3	2.4
1D + SNV	7	7	0.88	0.3	0.88	0.3	2.1

**Table 6:** PLSR modeling results based on the UVE feature screening.

Preprocessing Methods	Number of Characteristic Bands	Optimal Number of Principal Components	R_c_	RMSE_c_	R_p_	RMSE_p_	RPD
X	64	11	0.88	0.3	0.90	0.3	2.2
1D + MSC	23	8	0.93	0.3	0.93	0.3	3
1D + SNV	24	6	0.93	0.3	0.92	0.3	3

## Data Availability

The original contributions presented in this study are included in the article. Further inquiries can be directed to the corresponding author.
